# Hemophagocytic Syndrome in Children and Adults

**DOI:** 10.1007/s00005-014-0274-1

**Published:** 2014-02-09

**Authors:** Iwona Malinowska, Maciej Machaczka, Katarzyna Popko, Alicja Siwicka, Małgorzata Salamonowicz, Barbara Nasiłowska-Adamska

**Affiliations:** 1 Department of Pediatrics, Hematology and Oncology, Medical University of Warsaw, 00-576 Warsaw, Poland; 2 Division of Hematology, Department of Medicine at Huddinge, Karolinska Institutet, M54, SE-141 86 Stockholm, Sweden; 3 Medical Faculty, University of Rzeszow, Rzeszow, Poland; 4 Department of Laboratory Medicine and Pediatric Immunology, Medical University of Warsaw, Warsaw, Poland; 5 Institute of Hematology and Transfusiology, Warsaw, Poland

**Keywords:** Hemophagocytic lymphohistiocytosis, Hemophagocytic syndrome, Children, Adults

## Abstract

Hemophagocytic syndrome, also known as hemophagocytic lymphohistiocytosis (HLH), is a heterogenic syndrome, which leads to an acute, life-threatening inflammatory reaction. HLH occurs both in children and adults, and can be triggered by various inherited as well as acquired factors. Depending on the etiology, HLH can be divided into genetic (i.e., primary) and acquired (i.e., secondary) forms. Among genetic HLH forms, one can distinguish between familial HLH and other genetically conditioned forms of HLH. Acquired HLH can be typically triggered by infections, autoimmune diseases, and malignancies. The most common symptoms of HLH are unremitting fever, splenomegaly, and peripheral blood cytopenia. Some severely ill patients present with central nervous system involvement. Laboratory tests reveal hyperferritinemia (often >10,000 μg/L), increased serum concentration of soluble receptor α for interleukin-2 (>2,400 U/L), hypertriglyceridemia, hypofibrinogenemia, coagulopathy, hyponatremia, hypoproteinemia, and elevated liver transaminases and bilirubin. Prognosis in HLH is very serious. Genetic HLH is always lethal if adequate therapy is not administered. Similarly, severe acquired cases often lead to death without appropriate treatment. Since HLH can be encountered by various specialists in the medical field, basic knowledge of this entity such as diagnostic criteria and treatment should be familiar to all physicians.

## Introduction

Hemophagocytic syndrome, also known as hemophagocytic lymphohistiocytosis (HLH), is a state of severe, life-threatening inflammation caused by an excessive, prolonged, and ineffective immune response (Henter et al. 2007; Janka 2007).

The first description of HLH was published in 1952 and was then called familial hemophagocytic reticulosis (Farquhar and Claireaux 1952). In subsequent years, reports on familial and sporadic HLH cases were published by different groups (Arico et al. 1996; Henter et al. 1998; Reiner and Spivak 1988). Depending on the etiology, HLH can be divided into genetic (i.e., primary) and acquired (i.e., secondary: sHLH) forms (Henter et al. 2007; Janka 2009). An acquired HLH in children and adults develops as a consequence of intense immune activation caused by: (1) infection (infection-associated HLH: I-HLH), (2) autoimmune disease (A-HLH) or (3) cancer (malignancy-associated HLH: M-HLH) (Balwierz et al. 2010; Gupta and Weitzman 2010; Ishii et al. 2007; Janka 2009; Machaczka et al. 2011b; Rouphael et al. 2007). HLH may also occur in the course of certain metabolic diseases (e.g., Gaucher disease, lysinuric protein intolerance), immunosuppressive therapy, as well as after organ and stem cell transplantation (Abdelkefi et al. 2009; Machaczka et al. 2011a).

According to Henter et al. (1998), the incidence of HLH in children is 1.2 cases/million/year, but it is believed that these figures are strongly underestimated. An increasing number of HLH cases is recognized in Poland. Nevertheless, HLH is diagnosed in the Polish pediatric population less frequently (0.82/million/year) than in Western Europe, with a reasonable number of patients thought to be undiagnosed (own unpublished data).

The congenital form of the disease usually presents in infancy or early childhood (80 % of cases). However, the first episode of HLH can occur at any point in a patient’s life. Cases of primary HLH were reported in both fetuses and adults (Clementi et al. 2002; Jędrzejczak 2008; Malloy et al. 2004; Mougiakakos et al. 2012).

So far, most researches have been devoted to explaining the pathophysiology of genetic HLH. Perforin gene mutation as a cause of HLH was first described in 1999 (Stepp et al. 1999). Over the next years, several genes responsible for the development of genetic HLH were discovered (Arneson et al. 2007; Feldmann et al. 2003; zur Stadt et al. 2005, 2009). Their common feature is impairment in the production of proteins necessary for the proper functioning of granule cytotoxicity. In this process, cytotoxic granules containing perforin and granzyme are released into the immune synapse, which is formed between cytolytic cells [NK cells or cytotoxic T cells (CTLs)] and target cells (infected or malignant cells). Some of these proteins also play a role in the transport of other granules containing melanin for instance. Among genetic HLH, familial HLH (FHLH) and other genetically determined forms are distinguished (Bryceson et al. 2007; Henter et al. 2007; Horne et al. 2008). In FHLH, the only manifestation of the disease is hemophagocytic syndrome. Mutations in genes *PRF1* (FHLH-2), *UNC13D* (FHLH-3), *STX11* (FHLH-4) and *STXBP2* (FHLH-5) cause different subtypes of FHLH as listed in brackets (Feldmann et al. 2003; Stepp et al. 1999; zur Stadt et al. 2005, 2009). In other cases of genetic HLH, hemophagocytic syndrome is only one of the disease manifestations and does not necessarily be present (Weitzman 2011) (Table 1).

**Table 1:** Gene mutations and their impact on the development of HLH

Disease	Mutated gene abbreviation	Subsequent abnormality
FHLH-1	Unknown	Unknown
FHLH-2	*PRF1*	Lack of perforin or its abnormal function
FHLH-3	*UNC13D*	Abnormal vesicle priming and secretion of cytotoxic granules
FHLH-4	*STX11*	Abnormal vesicle intracellular trafficking and membrane fusion/docking of cytotoxic granules
FHLH-5	*STXBP2*	Abnormal vesicle intracellular trafficking and membrane fusion/docking of cytotoxic granules
Chédiak-Higashi syndrome	*LYST*	Abnormal melanin and cytolytic enzyme granule biogenesis
Griscelli syndrome 2	*RAB27A*	Abnormal docking of secretory granules
Hermansky-Pudlak syndrome type II	*AP3B1*	Abnormal intracellular trafficking of cytolytic granules
XLP1	*SH2D1A (SAP)*	Multiple abnormalities of cytotoxicity
XLP2	*BIRC4 (XIAP)*	Abnormalities in signal transduction in NK and CTLs/vesicle trafficking

According to Weitzman (2011)

Hemophagocytic syndrome may occur in very rare immune deficiency syndromes associated with albinism (i.e., Griscelli syndrome 2, Chédiak-Higashi syndrome, and Hermansky-Pudlak syndrome type II, which are caused by mutations in genes *RAB27A*, *LYST*, and *AP3B1*, respectively) and lymphoproliferative syndromes associated with chromosome X (XLP1 and XLP2, which are caused by mutations in genes *SH2D1A* and *BIRC4*, respectively) (Gupta and Weitzman 2010; Janka 2009). Other inherited conditions that can present with HLH are some known immunodeficiency syndromes such as X-linked SCID and X-linked hypogammaglobulinemia, Wiskott–Aldrich syndrome and DiGeorge syndrome del (22q11.2) (Arico et al. 1999; Pasic et al. 2003; Weitzman 2011). Recently, mutations in interleukin (IL)-2-inducible T cell kinase, CD27 and magnesium transporter gene have been reported to be associated with Epstein–Barr virus (EBV)-associated lymphoproliferation, lymphoma and HLH (Huck et al. 2009; Li et al. 2011; Stepensky et al. 2011; van Montfrans et al. 2012).

The cytotoxic activity of CTLs and NK cells plays a major role in the defense against viral infections and cancer. Cytotoxic cells exert their effect by releasing cytotoxic granules. This is a multistage process involving trafficking of cytotoxic granules containing perforin and granzyme toward the immune synapse, anchoring and connecting granules with the cell membrane, releasing the contents of granules and ultimately leading to death of the target cell through apoptosis (Gupta and Weitzman 2010; Janka 2007).

The pathogenesis of HLH is not fully understood. It is known, however, that the clinical symptoms are the results of excessive activation of CD8^+^ T lymphocytes and macrophages. An impaired ability of CTLs to remove antigen-presenting cells (APCs) leads to chronic stimulation of CD8^+^ cytotoxic lymphocytes and the release of cytokines. All clinical manifestations observed in HLH result from hypercytokinemia and the polyclonal proliferation of CTLs and APCs such as macrophages/histiocytes (Jordan et al. 2004; Komp et al. 1989). Infiltration by these cells and increases in tumor necrosis factor-α, IL-1β, IL-6, IL-8 and interferon-γ are responsible for the signs and symptoms of HLH (i.e., fever, hepatosplenomegaly, rashes, bleeding, jaundice, lymphadenopathy, changes in mental status, seizures, cranial nerve palsies) and abnormalities in laboratory studies (i.e., pancytopenia, hypofibrinogenemia, hypertriglyceridemia, hyperferritinemia, hypertransaminasemia, hyperbilirubinemia, pleocytosis in the cerebrospinal fluid, hypoalbuminemia and hyponatremia) (Chiang et al. 2013; Henter et al. 2007; Janka 2007).

Mechanisms of acquired HLH are likewise not completely understood. Unlike FHLH, most patients with sHLH do not present detectable abnormalities in the mechanisms of cytotoxicity. Recent studies in animal models suggest at least two different mechanisms of sHLH: (1) enhanced antigen presentation, and (2) excessive signaling of Toll-like receptors (Canna and Behrens 2012).

EBV infection, the major trigger of severe I-HLH, could be associated with the onset of FHLH (Imashuku et al. 1999a). Other infectious agents that can trigger HLH are cytomegalovirus, hepatitis C virus, varicella zoster virus, human herpesvirus (HHV)-6, HHV-8, HIV, influenza virus, rubella virus, parvovirus, adenovirus, *Mycobacterium tuberculosis*, Brucella species, *Treponema pallidum*, mycoplasma, parasites (e.g., leishmania) and invasive fungal infections (Janka 2009; Przybylski et al. 2010; Rouphael et al. 2007).

## Diagnosis of HLH

The most frequent signs of HLH are fever lasting ≥7 days, hepatosplenomegaly, pancytopenia, lymphadenopathy, and skin rashes occurring at presentation in most patients. In 2004, the Histiocyte Society developed the current diagnostic guidelines of HLH (Henter et al. 2007). According to these guidelines (HLH-2004), at least five of the following eight diagnostic criteria must be fulfilled for diagnosis: (1) fever, (2) splenomegaly, (3) cytopenia affecting ≥2 cell lines (hemoglobin <90 g/L, and in infants <100 g/L; platelet count <100 × 10^9^/L; neutrophils <1.0 × 10^9^/L), (4) hyperferritinemia (>500 μg/L), (5) hypertriglyceridemia (fasting triglycerides >3.0 mmol/L) and/or hypofibrinogenemia (<1.5 g/L), (6) hemophagocytosis in bone marrow, spleen, liver, or lymph nodes, (7) elevated level of soluble receptor α for IL-2 (sIL-2Rα, also called as sCD25) >2,400 U/mL, and (8) low or absent NK cell activity.

Of note, the presence of typical FHLH gene mutations, detected with molecular genetics modalities, is sufficient to establish a diagnosis, regardless of the number of fulfilled criteria according to the HLH-2004 (Henter et al. 2007).

Some genetic forms of HLH are characterized by specific clinical features. In patients with mutations in *STXBP2*, severe chronic inflammatory bowel disease (colitis) and hypogammaglobulinemia were frequently found (Ishii et al. 2005). Mutations in the Munc 13-4 (*UNC13D*) and Munc 18-2 (*STXBP2*) genes often cause abnormal platelet function, which causes a tendency to hemorrhage and bleeding during surgical procedures (Ishii et al. 2005; Meeths et al. 2011).

Crohn-like colitis has been reported in significant proportion of boys and men with a X-linked inhibitor of apoptosis (XIAP) deficiency caused by mutations in *BIRC4* (Speckmann et al. 2013). These patients often initially seek to the care of gastroenterologists because of the dominant symptoms. In the course of XLP, splenomegaly and recurrent self-limiting HLH are often observed (Yang et al. 2012). However, clinical phenotype of XIAP deficiency was not fully explained by the specific mutations of *BIRC4*, the protein expression levels, or the results of cell death studies (Speckmann et al. 2013).

## Interpretation of Test Results

Certain functional and numerical abnormalities of NK cells can be observed in the course of different forms of HLH.

The cytotoxic activity of NK cells can be determined by conducting a cytotoxic test and is measured by the number of K562 cells killed by cytotoxic cells. K562 cells are erythroleukemia cell line which is easily killed by NK cells as they lack the MHC complex required to inhibit NK activity. The test is performed using radioactive Cr or fluorescent dye (propidium iodide) and analyzed by flow cytometry. Impaired results can be observed in patients with genetically determined or secondary immune disorders. In most cases, they result from a reduction in the number of circulating NK cells or from the impairment in their cytotoxic function (Bryceson et al. 2010).

The results of a cytotoxic test could distinguish between patients with a reduced number of NK cells with preserved cytotoxic function, and patients with a normal or decreased number of NK cells with impaired cytotoxic ability. NK cell dysfunction is one of the diagnostic criteria of HLH.

In the familial forms of the disease, the cytotoxic response is impaired at different stages of cell activation, signal transduction, endocytosis, or perforin production. An aggressive viral infection in the case of sHLH can cause a reduction in the number of NK cells. In remission of sHLH, both the number and cytotoxic function of NK cells should return to normal. In the case of genetic defects, the disorder in cytotoxic function is persistent. Thus, when analyzing a patient showing a permanent reduction of NK cell activity while presenting a correct amount of these cells, one should consider the existence of the familial form of HLH (Bryceson et al. 2010).

Lysosome-associated membrane protein (LAMP)/CD107a is used as a marker for NK cell degranulation, but its role in NK cell biology remains unknown (Krzewski et al. 2013). Activation of NK cells is connected with the appearance of the cell surface antigens LAMP1/CD107a and LAMP2/CD107b, which are elements of the lysosomal membrane. Stimulation of NK cells leads to degranulation of cytotoxic granules, and displacement of CD107 molecules to the cell membrane. Identification of the exocytosis of LAMP1/CD107a and LAMP2/CD107b on the cell surface in the degranulation test indicates the activation of cytotoxic cells (Bryceson et al. 2012).

An impaired expression of the LAMP1/CD107a and/or LAMP2/CD107b antigens on the surface of NK cells indicates an impaired degranulation process and, finally, an impaired cytotoxic response. Such a result indicates the need for further genetic testing for primary HLH (Bryceson et al. 2007). Perforin and granzymes are released to the immune synapse as a result of lysosomal degranulation and are involved in the destruction of a target cell membrane in the cytotoxic response. Decreased production of perforin and granzymes, resulting from genetic mutations or inhibition of genes expression, leads to the reduction in NK cells’ cytotoxic capacity. Therefore, it is essential to complement NK cell function tests with a quantitative assessment of granzyme and perforin. Additional information could be provided by a quantitative expression assay of those genes responsible for the synthesis of these enzymes (mRNA) and the identification of mutations responsible for the occurrence of any quantitative disorders.

Mutations in *PRF1* gene (FHLH-2), which encodes perforin and is located on chromosome 10, are identified in 20–40 % FHLH patients. As a consequence, there is a defective production of perforin which is the component of cytotoxic cell granules responsible for the proper release of granzymes. Granzymes are proteolytic enzymes fragmenting DNA and causing apoptosis in target cells. Therefore, the consequence of mutations in *PRF1* gene is inhibition of cytotoxicity. Results of the functional tests reveal low cytotoxic activity but normal degranulation (Marsh et al. 2010a).

*UNC13D*, *STX11* and *STXBP2* genes are responsible for the production of proteins involved in the maturation and fusion of cytotoxic granules with the cell membrane (Bryceson et al. 2007; Chiang et al. 2013; Feldmann et al. 2003; Stepp et al. 1999; zur Stadt et al. 2005, 2009). Mutations in these genes impair the process of degranulation and secretion of perforin and granzymes. As a consequence, the cytotoxic function of NK cells and CTLs is inhibited. Aberrations in *UNC13D*, *STX11* and *STXBP2* can be identified from results of both the cytotoxicity assay and the degranulation test (Molleran Lee et al. 2004).

Increased concentration of the sIL-2Rα (sCD25) is a measure of T cell activation (Chiang et al. 2013; Komp et al. 1989). However, an assay determining a serum concentration of sCD25 is carried out only in a few specialized laboratories.

**Fig. 1:**
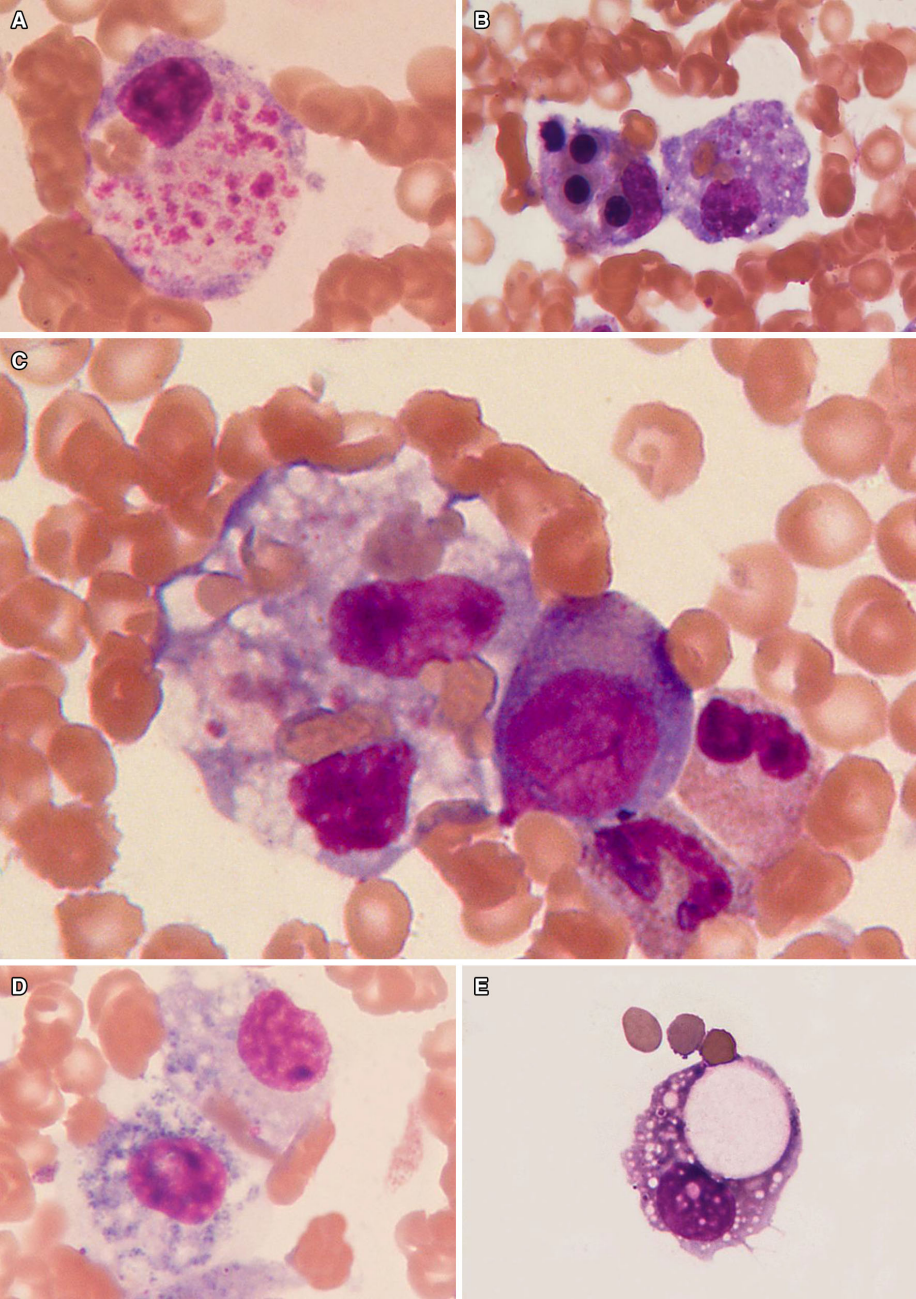
Hemophagocytosis present in bone marrow (**a**–**d**) and cerebrospinal fluid (**e**) smears in the course of HLH in humans. Activated macrophages show intense hemophagocytosis of different hematopoietic cell lines

The presence of hemophagocytosis (Fig. 1) is only one of the eight diagnostic criteria of HLH (Henter et al. 2007). Hemophagocytosis takes place through the heme-binding receptor CD163 on the activated macrophages, and increased serum concentration of the soluble CD163 (sCD163) is a sign of macrophage activation. However, one should remember that this morphological hallmark of HLH is quite often absent in the bone marrow or lymph nodes, especially in the beginning of HLH.

## HLH in Adults

Literature on the topic of HLH in adults is limited. This is partially due to the ambiguity of terminology [e.g., macrophage activation syndrome (MAS) vs. A-HLH] (Ramanan and Baildam 2002), the lack of homogeneity of diagnostic criteria used by different authors (Kumakura 2005), and finally, the lack of systematic research focusing on the issue of HLH in adults. All acquired forms of HLH are often, similarly to FHLH, triggered by infection (commonly by viral infections such as EBV), which can sometimes lead to difficulties in the proper classification of HLH (Janka 2009; Rouphael et al. 2007). Moreover, the problem of HLH in adults is hardly recognized by physicians not involved in research on HLH and, therefore, often goes unrecognized (Janka 2009; Jędrzejczak 2008).

In adults, acquired forms of HLH are the most prevalent (Ishii et al. 2007). This patient group can develop HLH as a result of immunological activation due to infection (I-HLH), autoimmune disorders (A-HLH/MAS), or malignancies (M-HLH) (Balwierz et al. 2010; Gupta and Weitzman 2010; Ishii et al. 2007; Janka 2009; Machaczka et al. 2011b; Rouphael et al. 2007).

A retrospective study from Japan revealed that the frequency of different forms of HLH in adults varied depending on the age bracket (Ishii et al. 2007). Among HLH patients aged 15–29 years, I-HLH was the most common (68 % of cases). It was caused in equal parts by EBV-HLH and I-HLH other than EBV-HLH. In this age group, the second most common cause of HLH was A-HLH (22 %), followed by the M-HLH (10 %) (Ishii et al. 2007). In patients aged 30–59 years, M-HLH occurred almost as often as I-HLH (37 vs. 47 %, respectively), followed by A-HLH (9 %) and HLH after hematopoietic stem cell transplantation (post-SCT HLH) (7 %). Importantly, in the group of patients aged ≥60 years, however, M-HLH was the most frequent (68 %), followed by the I-HLH (26 %, caused mostly by infection other than EBV) and A-HLH (6 %) (Ishii et al. 2007).

Epidemiological data on the prevalence of HLH among adults in the European population are very limited. A retrospective Swedish study showed the annual incidence of M-HLH in adults, in south-western Sweden, to be 1:280,000 inhabitants per year or 0.36/100,000 inhabitants per year, which indicates a relatively high incidence of M-HLH in adults (Machaczka et al. 2011b). This finding should be treated with caution due to the small patient population, although a long observation period of over 14 years increases the reliability of the study. Moreover, studies at the Karolinska University Hospital in Stockholm in recent years confirm that M-HLH among adults (particularly with hematological malignancies) is much more prevalent than previously believed (Löfstedt et al. 2013).

M-HLH develops most frequently in patients with T- and NK-cell lymphoma, but it can also occur in the course of other hematological malignancies (e.g., B-cell lymphoma, Hodgkin’s lymphoma, multiple myeloma, myelodysplastic syndromes, acute and chronic leukemias) and solid cancers (e.g., thymoma, germ cell tumor, carcinoma, and hepatocellular carcinoma) (Balwierz et al. 2010; Hasselblom et al. 2004; Ishii et al. 2007; Janka 2009; Machaczka and Vaktnäs 2007; Machaczka et al. 2010, 2011b, 2011c, 2012; Reiner and Spivak 1988; Tong et al. 2008). Ishii and colleagues (2007) reported that in Japan the causes of 18 % (24/132 patients) of M-HLH cases were cancers other than lymphoma. Among them, there were nine cases of HLH associated with acute myeloid leukemia and three cases of HLH associated with myelodysplastic syndromes. Of note, it is worth remembering that M-HLH can occur before or during treatment of a known malignancy, or as the first manifestation of an occult malignancy (Janka 2009).

Clinical signs observed in M-HLH are similar to those typical for FHLH (Henter et al. 2007; Machaczka et al. 2011b). Difficulties in the rapid diagnosis of M-HLH result from the fact that typical HLH symptoms, such as persistent fever, splenomegaly and cytopenia, are not specific. The characteristic laboratory findings are hyperferritinemia (often “sky high” >10,000 μg/L), hypertriglyceridemia, hypofibrinogenemia, liver function tests abnormalities (i.e., elevated transaminases and bilirubin), coagulopathy (inclusive disseminated intravascular coagulation), hyponatremia, elevated serum lactate dehydrogenase, and hypoproteinemia (Hasselblom et al. 2004; Henter et al. 2007; Machaczka et al. 2011b; Tong et al. 2008). Involvement of the central nervous system (CNS) may be present in severe forms of M-HLH (Henter et al. 2007; Machaczka et al. 2011b, 2012). The clinical criteria of FHLH can be applied for the diagnosis of secondary HLH other than FHLH (Henter et al. 2007).

Every immunosuppressive therapy may predispose one to the development of HLH. Therefore, the possibility of HLH should also be considered in patients in remission of a malignancy who underwent autologous or allogeneic stem cell transplantation (Abdelkefi et al. 2009; Machaczka et al. 2011c, 2012).

M-HLH has the worst outcome of all the different HLH forms (Ishii et al. 2007; Machaczka et al. 2011b; Shabbir et al. 2011; Tong et al. 2008). In a Japanese study, the reported 5-year overall survival rate was only 12 % in patients with HLH associated with T/NK-cell lymphoma and 48 % in HLH associated with B-cell lymphoma (Ishii et al. 2007). For the sake of comparison, in the same study, the 5-year survival rate in patients with I-HLH or A-HLH was 83–90 %, and 54 % in patients with FHLH. Tong et al. (2008) published data concerning the outcome of 28 patients suffering from aggressive T-cell lymphoma, presenting at diagnosis with concomitant HLH. All 28 patients died; 89 % (25/28) died within 6 months of diagnosis, 7 % (2/28) died 6–12 months after diagnosis, and one patient died 22 months after diagnosis of T-cell lymphoma and HLH. The median survival time was only 40 days (range 16 days to 22 months) for the entire group (Tong et al. 2008).

In our experience, the clinical course of M-HLH in adults was aggressive in all cases (Machaczka and Vaktnäs 2007; Machaczka et al. 2010, 2011a, 2011b, 2011c, 2012). Although in some patients with M-HLH, a poor outcome depends on the progression of underlying malignancy, in some patients the lack of effective M-HLH therapy may further impede the adequate treatment of their malignancy (Ishii et al. 2007; Janka 2009; Machaczka et al. 2011b).

## Prognosis and Treatment

Before the introduction of modern HLH treatment modalities, the 1-year survival in FHLH was close to 0 % (Arico et al. 1996). After introduction of the HLH-94 protocol, overall survival increased to 55 % in acquired HLH and to 62 % in FHLH additionally treated by means of HLA-matched allogeneic bone marrow transplantation (Henter et al. 2002). Currently, after the introduction of reduced intensity conditioning (RIC) before allogeneic hematopoietic stem cell transplantation (allo-SCT), overall survival in FHLH has reached 92 % (Marsh et al. 2010b). However, the mortality rate of children with acquired HLH still ranges between 8 and 22 %.

Treatment of HLH is difficult, long-lasting and often associated with a high morbidity and mortality (Henter et al. 2007; Janka 2009). The therapy of any form of HLH in children and adults should focus on suppression of the hyperactivated immune system by destruction of activated CD8^+^ T lymphocytes and macrophages, and treatment of any existing HLH triggers (Janka 2009). In cases of FHLH, an additional aim is correction of the underlying immune defect (Henter et al. 2007).

Therapy based on the HLH-94 protocol, proven to be effective in the treatment of FHLH, proves to be a reasonable option in adults with M-HLH in comparison with other approaches, including symptomatic treatment. However, the overall response rate in adults with M-HLH was 50 %, thus a further search for more effective treatment is necessary (Machaczka et al. 2011b). In our experience, active infection complicating the course of M-HLH may lead to the development of fulminant HLH and ultimately, death. Therefore, prophylaxis and early treatment of infections seem to be reasonable in the course of HLH. Allo-SCT might have a curative potential not only for FHLH, but also for refractory cases of acquired HLH, such as EBV-HLH (Henter et al. 2007; Horne et al. 2005; Jordan and Filipovich 2008; Ohga et al. 2010). Allo-SCT performed to consolidate remission of malignancy and HLH may have a curative impact on both entities (Machaczka et al. 2012). Therefore, when discussing possible treatment options for patients with M-HLH, allo-SCT should be considered in eligible individuals.

The first use of allogeneic multipotent mesenchymal stromal cells (MSCs) in the therapy of refractory FHLH in a young adult suggests that this approach may achieve the desired immunosuppressive effects (Mougiakakos et al. 2012). Moreover, the aforementioned results suggest that the use of MSCs can in some cases help to avoid the use of cytotoxic drugs to control exacerbations of HLH. One can also speculate that the use of MSCs will support the patient long enough to allow an effective bridge to allo-SCT in patients with FHLH, i.e., to search for a suitable stem cell donor.

Autoimmune-associated HLH, despite proposals to harmonize terminology often referred to by many rheumatologists as MAS, has most often been reported in the systemic form of juvenile idiopathic arthritis (sJIA; 7–10 % of patients with sJIA) (Canna and Behrens 2012; Gupta and Weitzman 2010; Janka 2007, 2009). A-HLH can also occur in the course of other autoimmune diseases. However, information about A-HLH in adults is very limited (Machaczka et al. 2011d). A-HLH in adults may present as an aggressive and life-threatening disease (Gupta and Weitzman 2010; Janka 2009; Machaczka et al. 2011d). Signs and symptoms are often difficult to distinguish from an exacerbation of the patient’s underlying autoimmune disease, which increases the risk of delaying diagnosis and appropriate therapy. Treatment based on the protocol HLH-94 may favorably affect the outcome in severe cases of A-HLH in adults (Machaczka et al. 2011d).

The utility of biological response modifiers in the therapy of HLH remains unclear. The use of monoclonal antibodies such as anti-CD52 (Campath) in acquired HLH has produced conflicting results. Our experience indicates that anti-CD52 may induce HLH in patients suffering from hematological malignancy (Machaczka et al. 2010).

## Allogeneic Stem Cell Transplantation for HLH

The first successful allogeneic bone marrow transplantation performed for the treatment of HLH was reported by Fischer et al. (1986). Since then, information regarding the role of allo-SCT in the therapy of HLH has mostly concerned FHLH. Immunochemotherapy (e.g., HLH-94 and HLH-2004 protocols) is only temporarily efficient in the control of FHLH, and the outcome is uniformly lethal unless the patient undergoes allo-SCT (Henter et al. 2007; Jordan and Filipovich 2008).

In cases of FHLH, different groups of researchers have reported 5-year overall survival rates of 50–70 % with myeloablative conditioning (Baker et al. 1997; Cesaro et al. 2008; Durken et al. 1999; Horne et al. 2005; Imashuku et al. 1999b; Ouachèe-Chardin et al. 2006), and 75–92 % with RIC (Cooper et al. 2006; Marsh et al. 2010b). However, early treatment-related mortality (TRM) occurring within the first 100 days following allo-SCT remains the major obstacle to the cure of FHLH by allo-SCT (Baker et al. 1997; Cesaro et al. 2008; Horne et al. 2005; Ouachèe-Chardin et al. 2006). Infections, veno-occlusive disease (VOD), pneumonitis, graft failure, and graft-versus-host disease have been reported as the main causes of early TRM (Baker et al. 1997; Cesaro et al. 2008; Cooper et al. 2006; Durken et al. 1999; Horne et al. 2005; Imashuku et al. 1999b; Marsh et al. 2010b; Ouachèe-Chardin et al. 2006). TRM may depend on the unsatisfactory control of HLH before allo-SCT in some patients. Thus, every possible effort must be made to ensure that patients proceed to allo-SCT with optimal control of their underlying FHLH (Jordan and Filipovich 2008). Of note, systemic and CNS manifestations of HLH should be carefully monitored and treated while preparing for allo-SCT. However, significant excess mortality has also been seen in patients with apparently good control of their underlying FHLH before allo-SCT (Jordan and Filipovich 2008). It is possible that occult liver or lung damage from HLH may predispose FHLH patients to high rates of VOD or pneumonitis when treated with a busulfan-based conditioning regimen. Moreover, a significant number of early deaths (≤100 days after allo-SCT) are attributed to HLH reactivation (Filipovich et al. 2010). On the other hand, patients who survived >100 days after allo-SCT with durable engraftment usually experienced long-term survival free of HLH (Cesaro et al. 2008; Ohga et al. 2010).

Allogeneic hematopoietic stem cell transplantation has been performed only occasionally in cases of acquired HLH. Rare case reports have previously been published on refractory EBV-HLH successfully treated by means of allo-SCT (Minegishi et al. 2001; Sovinz et al. 2010; Toubo et al. 2004). However, a recent Japanese study disclosed a curative effect of allo-SCT in 64 % (7/11) of patients with refractory, life-threatening EBV-HLH (Ohga et al. 2010). In the same year, Yoon et al. (2010) reported in their study that allo-SCT could be a curative therapy for children with relapsed/refractory acquired HLH. Anecdotal reports have also shown the efficacy of allo-SCT for M-HLH (Chang et al. 2009; Goi et al. 1999; Kelly et al. 2011; Machaczka et al. 2012). Of note, the role of donor lymphocyte infusion in therapy of M-HLH treated by means of allo-SCT remains to be established.

The best results following myeloablative conditioning allo-SCT for FHLH have been achieved when HLA-matched related or unrelated donors have been used, and CNS disease has been absent or quiescent at the time of allo-SCT (Jordan and Filipovich 2008). Additionally, when searching for a suitable donor for allo-SCT, the high incidence of HLH following umbilical cord blood transplantation in adults, reported by Takagi et al. (2009), should be taken into consideration.

In conclusion, HLH is a heterogeneous syndrome of hyperinflammation caused by genetic and acquired factors. The pathogenesis of acquired forms of HLH is not fully understood and it requires further studies. Due to the acute and life-threatening course of the disease, awareness of HLH’s signs and symptoms as well as diagnostic criteria should be mandatory among all physicians, since early diagnosis and prompt introduction of adequate therapy are crucial for its outcome. It is worth pointing out that M-HLH can occur before or during the treatment of known malignancy, or as the first manifestation of an occult malignancy. Implementation of allo-SCT in the therapy of relapsed/refractory cases of acquired HLH should be further investigated, in hope to achieve better treatment results in children and adults. Last but not least, immunomodulatory therapy with allogeneic transplantation of MSCs may represent a beneficial approach for some patients with FHLH or acquired HLH.
